# Costunolide prevents renal ischemia-reperfusion injury in rats by reducing autophagy, apoptosis, inflammation, and DNA damage

**DOI:** 10.22038/IJBMS.2023.71779.15596

**Published:** 2023

**Authors:** Mustafa Can Güler, Erol Akpinar, Ayhan Tanyeli, Selim Çomakli, Yasin Bayir

**Affiliations:** 1 Department of Physiology, Faculty of Medicine, Atatürk University, Erzurum, Turkey; 2 Department of Pharmacology, Faculty of Medicine, Atatürk University, Erzurum, Turkey; 3 Department of Pathology, Faculty of Veterinary, Atatürk University, Erzurum, Turkey; 4 Department of Biochemistry, Faculty of Pharmacy, Atatürk University, Erzurum, Turkey

**Keywords:** Apoptosis, Autophagy, Costunolide, Rat, Renal ischemia-reperfusion

## Abstract

**Objective(s):**

Renal ischemia-reperfusion (I/R) is a vital health condition leading to acute kidney injury. Costunolide (COST) is an actively used molecule clinically for its anti-inflammatory, antioxidant, and immunomodulatory properties. In the present study, we searched for the possible protective effects of COST against renal ischemia/reperfusion (I/R) injury in rats.

**Materials and Methods:**

We established a renal I/R rat model. We divided forty rats into four groups: group I (sham), group II (I/R), group III (I/R+COST 5 mg/kg), and group IV (I/R+COST 10 mg/kg). We collected blood, kidney, and lung samples for analysis.

**Results:**

COST administration performed anti-oxidant and anti-inflammatory activity by reducing oxidant parameters and proinflammatory cytokine levels. COST alleviated DNA damage through declining 8-hydroxydeoxyguanosine (8-OHdG) levels. In addition, COST diminished tubular damage and inflammation by reducing kidney injury molecule-1 (KIM-1) production. COST administration also ameliorated apoptosis and autophagy by decreasing caspase-3 and microtubule-associated protein light chain 3B (MAPLC3, LC3B) expression.

**Conclusion:**

COST demonstrated protective effects against renal I/R-induced injury.

## Introduction

Acute kidney injury (AKI) is a lethal medical condition (1). An inflammatory response occurs during AKI, and proinflammatory cytokines like interleukin-6 (IL-6), interleukin-1β (IL-1β), and tumor necrosis factor-α (TNF-α) are produced (2). Renal ischemia-reperfusion (I/R) injury is the etiology of AKI (3). Several conditions, including renal transplantation (4), nephrectomy (5), hypotension (6), and severe allergic reactions (7), may lead to renal I/R injury.

Oxidative stress plays a vital role in the formation of renal I/R injury. One of the most critical and expected consequences of oxidative stress is peroxidation (8). Malondialdehyde (MDA) indicates prolonged cell damage and oxidative stress (9). Myeloperoxidase (MPO) is a peroxidase enzyme located in the granules of neutrophils and monocytes. Reperfusion-induced macrophage influx elevates MPO levels (10). 

Reactive oxygen species (ROS) are critical factors in kidney damage development and harm many substances, such as enzymes, proteins, and lipids (11). ROS from renal reperfusion can worsen oxidative stress, inflammation, apoptosis, and acute renal failure (12). Anti-oxidant mechanisms involve several enzymes like glutathione (GSH) and superoxide dismutase (SOD) coping with the detrimental effects of ROS (13). They decline renal I/R damage by reducing excessive ROS production (14).

Excess ROS production induces apoptosis. Caspases, including caspase 3, are activated during apoptosis and are the essential mediators of the apoptotic process (15). In addition to ROS, inflammatory response promotes neutrophil recruitment, proinflammatory cytokine generation (IL-1β, TNF-α, IL-6, etc.), and apoptosis (16). 

Autophagy is a physiological process that induces intracellular components to maintain intracellular homeostasis (17). However, uncontrolled autophagy eventually leads to cell death and may contribute to I/R damage (18). Microtubule-associated protein light chain 3B (MAPLC3, LC3B) is one of the most widely used autophagy markers (19). Localization of LC3B in the cytoplasm with lysosomes is preferred to evaluate the degree of autophagy (20).

8-hydroxydeoxyguanosine (8-OHdG) is a principal deoxyribonucleic acid (DNA) oxidation product. The cellular 8-OHdG concentration measures oxidative stress (21), and thus, it is preferred as an oxidative DNA injury biomarker (22). In the presence of I/R injury, 8-OHdG levels elevate (23). 

Kidney injury molecule-1 (KIM-1) is a type 1 transmembrane glycoprotein. It cannot be detected in normal kidney tissue but can be induced in ischemic and toxic conditions (24). Tubular epithelial cells are the most vulnerable parts in I/R-induced AKI (25). KIM-1 is highly expressed in the proximal tubule in response to renal ischemia (26). KIM-1 concentration corresponds to the severity of tubular damage (27). Increasing KIM-1 levels also suggest a rise in tubular inflammation (28).

Ischemic AKI usually activates immune responses such as proinflammatory cytokine regulation and pulmonary microvascular barrier disruption, resulting in lung inflammation, programmed cell death, and caspase activation (29). Increasing evidence suggests that pulmonary cellular apoptosis following renal I/R injury may play a key role in acute lung injury (30).

Costunolide (COST) is a natural sesquiterpene lactone (STL) commonly found in many plant families. Many herbs containing COST are used in traditional North Asian pharmacology for inflammatory and infectious diseases. In this context, studies investigating the anti-inflammatory, anti-oxidant, and immunomodulatory activities of COST have been conducted (31, 32). Antiallergic, neuroprotective, hepatoprotective, anticancer, and antidiabetic properties of COST have also been reported (33). 

We have not found any study in the literature related to the effects of COST on kidney I/R injury. In the current study, we aimed to investigate some oxidant-anti-oxidant values and molecular and histopathological parameters related to inflammatory, autophagic, and apoptotic processes to evaluate the effects of COST in the renal I/R injury model in rats.

## Materials and Methods

We carried out the present study within the laboratories of Atatürk University Medical Experimental Application and Research Center (MEARC), Faculty of Medicine, Faculty of Veterinary Medicine, and Faculty of Pharmacy.

Atatürk University Local Ethics Council of Animal Experiments approved the study (26.06.2020/96). We performed the study in compliance with the existing protocols of the ethics committee and the Helsinki Declaration of the World Medical Association recommendations on animal studies.

***Experimental animals***

We supplied forty 12-16 weeks old, 200-250 g, Wistar albino male rats from MEARC. During the experiment, we individually housed the animals in standard laboratory conditions (20-22 ^°^C temperature, 55% humidity, 12 hr day/night cycle) and 470x312x260 mm sized cages. We allowed them to get used to the environment for ten days before the study. The night before the I/R procedure, we did not feed them but allowed them tap water. 

***The administration of COST***

We used the COST hormone originating from Medchem Express (USA). We kept COST under suitable conditions (-20 ^°^C, sealed storage, away from moisture and light) until the moment of application. We have established the COST doses (5 mg/kg and 10 mg/kg) to be used intraperitoneal** (**IP) during the renal I/R model based on and modified from previous animal studies (34). 

***Surgical procedure***

Bilateral clamping of the renal arteries is frequently used to induce ischemia in animals; because this model correlates with the pathophysiological conditions of AKI in humans, in which impaired blood flow damages the kidneys (35). Considering the above criteria, we created a renal I/R model by clamping the bilateral renal arteries.

For the experiment, the animals were fixed in the prone position. After the back was shaved, it was disinfected with 10% povidone-iodine. Before all surgical procedures, we administered IP anesthesia with 100 mg/kg of ketamine and 15 mg of xylazine (36).

We designed the bilateral renal I/R model based on a previous model in the literature (37). Side incisions were made to reveal the left and right kidneys. We created bilateral ischemia using non-traumatic microaneurysm clamps to block both renal pedicles. We visually confirmed complete ischemia with a red-to-dark purple color change in the kidney within a few seconds. We carefully released the clamps after the 45 min ischemic period to begin reperfusion for 24 hr. We observed for 2-5 min until the kidneys returned to their original color to confirm that the blood flow was providing adequate restoration. We then closed the incisions with continuous sutures in two layers.

***Experimental groups***

We created four randomized experimental groups of ten animals each (n=10):

Group I (Sham): The dorsal regions of the animals were opened and closed without any procedure. 

Group II (I/R): The dorsal region of the rats was opened, and the renal arteries were clamped bilaterally. Following 45 min of ischemia, the clamps were removed, and reperfusion was performed for 24 hr.

Group III (I/R+COST 5 mg/kg): The surgical procedures applied in group II were repeated. Animals were administered IP 5 mg/kg COST 30 min prior to reperfusion.

Group IV (I/R+COST 10 mg/kg): All steps were the same as in group III, only the COST dose was 10 mg/kg.

After 24 hr of reperfusion, we sacrificed the animals with high-dose anesthesia. We took lung, kidney, and blood samples for molecular, oxidative, and histopathological examinations. 

***Biochemical analysis***

We ground some lung and kidney tissue samples with liquid nitrogen and homogenized them for molecular and oxidative analyses. The oxidative analyses (MPO, SOD, GSH, and MDA) were performed by calorimetric methods (38-40). 

We stored the serum obtained by centrifuging blood samples in a deep freezer at -80 ^°^C for biochemical measurements. IL-10, IL-1β, TNF-α, and IL-6 (BT LAB, Cat No: E0108Ra, Cat No: E0108Ra, Cat No: E0764Ra, and Cat No: E0764Ra) values were measured with an ELISA reader (ELISA, BioTEK PowerWave XS Winooski, UK). 

***Histopathological procedures***

*Light microscopy process*

The lung and kidney tissue samples were obtained and fixed in a 10% buffered formaldehyde solution following the experiment. Then, formaldehyde in the tissues was removed, and the tissues were set in paraffin. A microtome (Leica RM2255/UK) was used to cut 5 μm sections from paraffin blocks on slides.

***Hematoxylin-eosin staining protocol***

After deparaffinization, sections were subjected to an alcohol series. Hematoxylin-Eosin (HE) was then used to stain the tissue samples. Kidney tissue samples were evaluated for hemorrhage and necrotic changes, and lung tissue samples were examined for pulmonary interstitial edema, inflammatory cell infiltration, and bleeding under a light microscope (Olympus BX51/DP72).

***Immunohistochemical staining protocol***

Deparaffinized and rehydrated tissue sections were taken into citrate buffer solution (pH 6.0) and heated in the microwave at 800 W (10 min) for antigen retrieval. Tissue sections were incubated at 37 ^°^C for one hour with Caspase-3, 8-OHdG, LC3B, and KIM-1 antibodies. The sections were then thoroughly rinsed three times with saline and incubated for 30 min at room temperature with a secondary antibody (Ultra Vision Large Volume Detection System; TP-125-HL; Lab Vision, Thermo). Then, 3’3 diaminobenzidine (DAB) was applied to treat the sections, and hematoxylin was administered for counterstaining. The findings were analyzed with a DP72 Microscope (Olympus, Inc., Tokyo, Japan). Positivity was scored as none:-, mild:+, moderate:++, and intense:+++.

***Statistical analysis***

For statistical analysis, we utilized SPSS 20.0 software (IBM Corp, Armonk, NY, USA). The findings were displayed as mean±standard deviation. One-way ANOVA and Duncan’s multiple comparison tests were used to compare the groups. A *P*-value<0.05 was regarded as statistically significant. Means written with the same letter in the same column are not statistically different in the Duncan test.

We used the GraphPad (Prism Software Inc., Version 7.0, California, USA) program for the statistical evaluation of histopathological data. We applied Kolmogorov-Smirnov test for normal distribution between groups, and used nonparametric tests for histopathology and immunohistochemical analyzes that were not normally distributed. The difference between groups was determined using the Kruskal-Wallis test in the histopathological examination. We preformed the Mann-Whitney U test to determine the groups that made the difference. A *P*-value<0.05 was considered statistically significant.

## Results

***Biochemical findings***

*IL-10, IL-6, IL-1β, and TNF-α levels*

**Figure 1:**
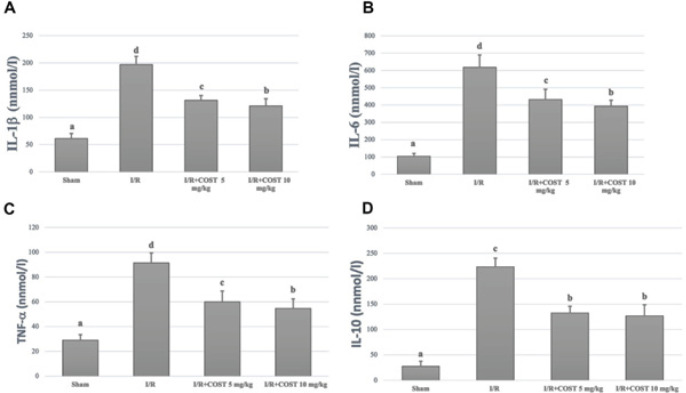
Serum IL-1β, IL-6, TNF-α, and IL-10 levels in the experimental groups of renal ischemia reperfusion rat model

Figure 1 shows serum IL-10, IL-6, IL-1β, and TNF-α levels. The parameters elevated statistically meaningfully in the I/R group compared to the sham group (*P*<0.05). There were substantial reductions in the low and high-dose (COST 5 mg and 10 mg) groups compared to the I/R group (*P*<0.05). When the COST groups were compared, the decline in IL-1β, TNF-α, and IL-6 levels in the high-dose COST group was more significant than in the low-dose COST group (*P*<0.05). IL-10 levels did not differ between the COST groups.

*MDA, MPO, SOD, and GSH levels*

**Figure 2:**
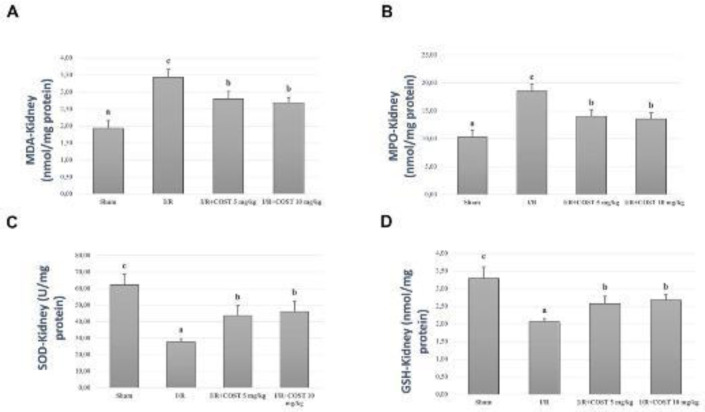
Kidney MDA, MPO, SOD, and GSH levels in the experimental groups of renal ischemia reperfusion rat model

**Figure 3:**
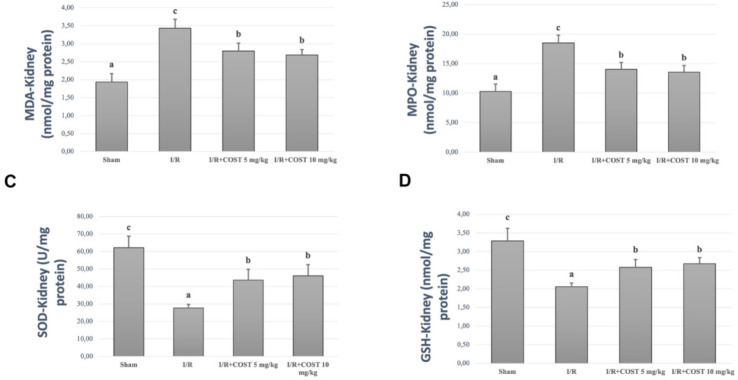
Lung MDA, MPO, SOD, and GSH levels in the experimental groups of renal ischemia-reperfusion rat model

Figures 2 and 3 show MDA, MPO, SOD, and GSH measured in kidney and lung tissue samples. I/R group values were significantly higher than those of the sham group (*P*<0.05). There was a significant decrease in the low and high-dose COST groups compared to the I/R group (*P*<0.05). However, COST groups did not differ significantly. 

***Histopathological findings***

**Figure 4:**
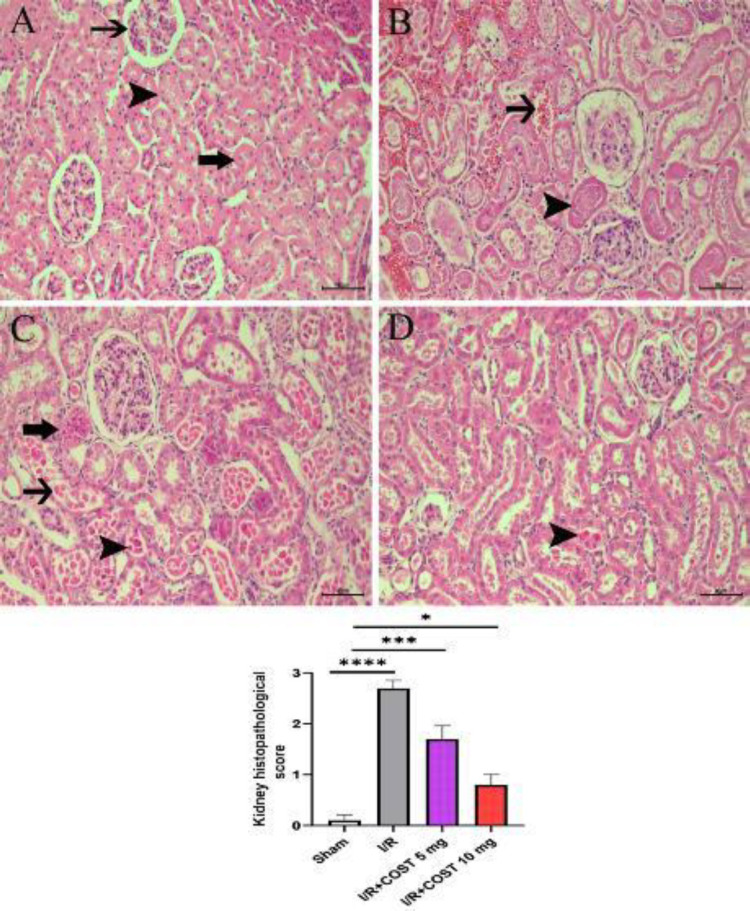
Histopathological changes and the histopathological score of kidney tissues of renal ischemia-reperfusion rat model

Figure 4 shows the histopathological findings and histopathological score of the kidney tissues in the study. No histopathological changes were observed in the kidney tissue of rats in the sham group (Figure 4A). In the I/R group, degeneration in the proximal and distal tubules and epithelial cells with dense pycnotic and karyolitic nuclei was observed. Intense necrosis was also found in the glomerular tuft. The most affected area was the cortical and corticomedullary border. The lumens of the affected tubules contained exfoliated necrotic cells and granular eosinophilic remnants. Extensive bleeding was observed in the intertubular space (Figure 4B). 

Although degenerative and necrotic changes decreased in the I/R+COST 5 mg/kg group, there were pycnotic and karyolitic cell groups in the tubule epithelium and lumens. Bleeding was quite less compared to the I/R group (Figure 4C). Tubular epithelial cells with degenerative and necrotic nuclei were significantly reduced in the I/R+COST 10 mg/kg group compared to the I/R and I/R+COST 5 mg/kg groups (Figure 4D).

**Figure 5:**
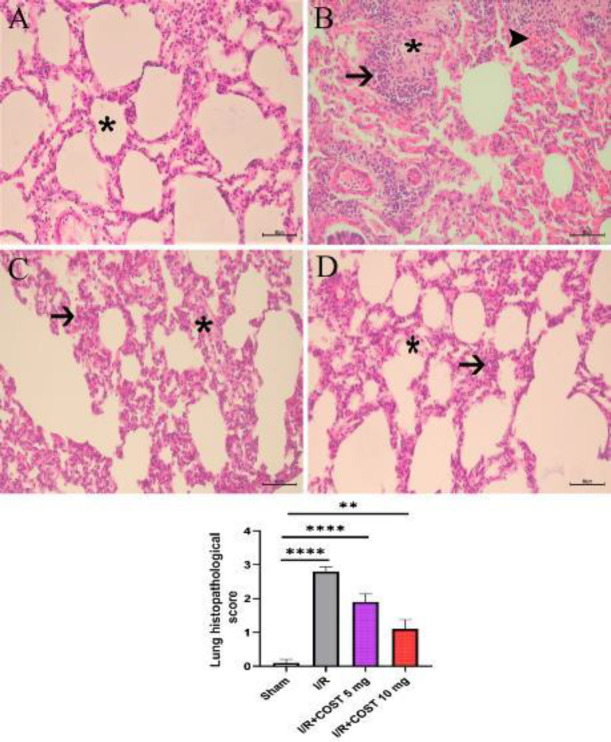
Histopathological changes and histopathology score of the lung tissues of renal ischemia-reperfusion rat model

The histological findings of the lung tissues and the histopathological score are shown in Figure 5. The sham group’s alveolar space and lung tissue were in good condition. There was no sign of hemorrhage, inflammatory cell infiltration, or edema in the alveolar septum (Figure 5A). In the I/R group, hemorrhage, severe edema in the interstitial tissue and alveolar septum, and significant inflammatory cell infiltration all took place (Figure 5B). The I/R+COST 5 mg/kg group had moderate inflammatory cell infiltration and alveolar septal edema (Figure 5C). Compared to the I/R and I/R+COST 5 mg/kg groups, the I/R+COST 10 mg/kg group had less inflammation and edema, and the alveolar opening had begun to resemble normal (Figure 5D).

***Immunohistochemical findings***

Immunohistochemical staining was used to assess the expressions of KIM-1, 8-OHdG, caspase-3, and LC3B in tissue samples. 

***KIM-1***

**Figure 6:**
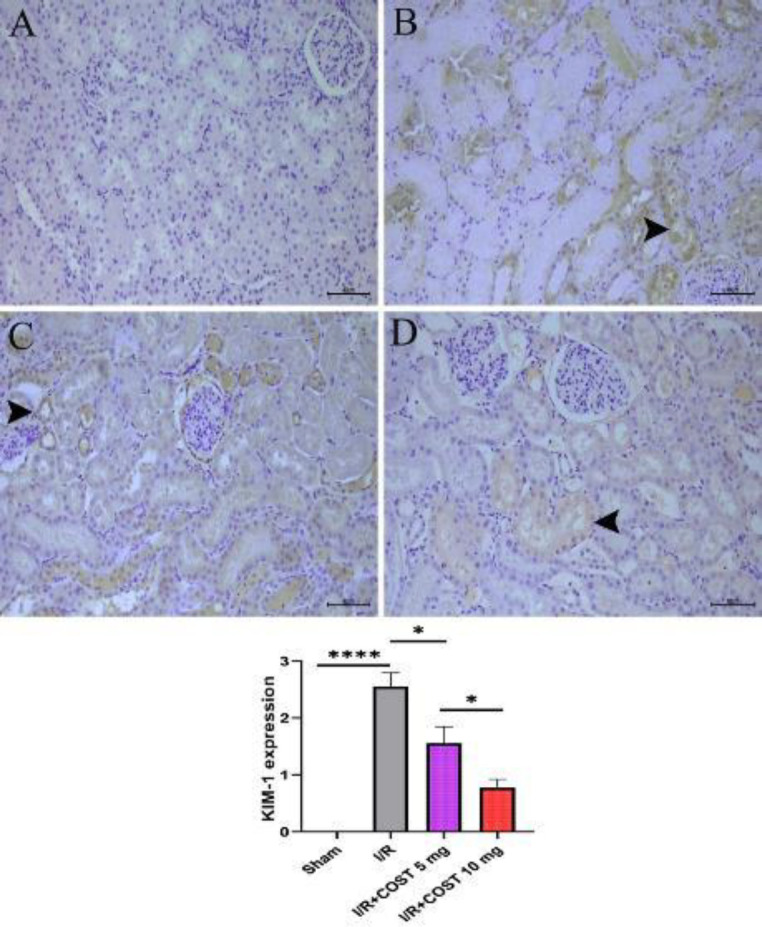
KIM-1 expression and KIM-1 expression score of kidney tissues of renal ischemia-reperfusion rat model

Figure 6 illustrates KIM-1 expression in kidney tissues together with KIM-1 expression scores. There was no KIM-1 expression in the sham group (Figure 6A). In the I/R group, renal tubular epithelial cells and interstitial cells had the most significant KIM-1 expression (Figure 6B). KIM-1 expression was markedly decreased following I/R+COST 5 mg/kg therapy (Figure 6C). KIM-1 expression was considerably lower in the I/R+COST 10 mg/kg group than in the I/R+COST 5 mg/kg group (Figure 6D)

***8-OHdG (Kidney)***

**Figure 7:**
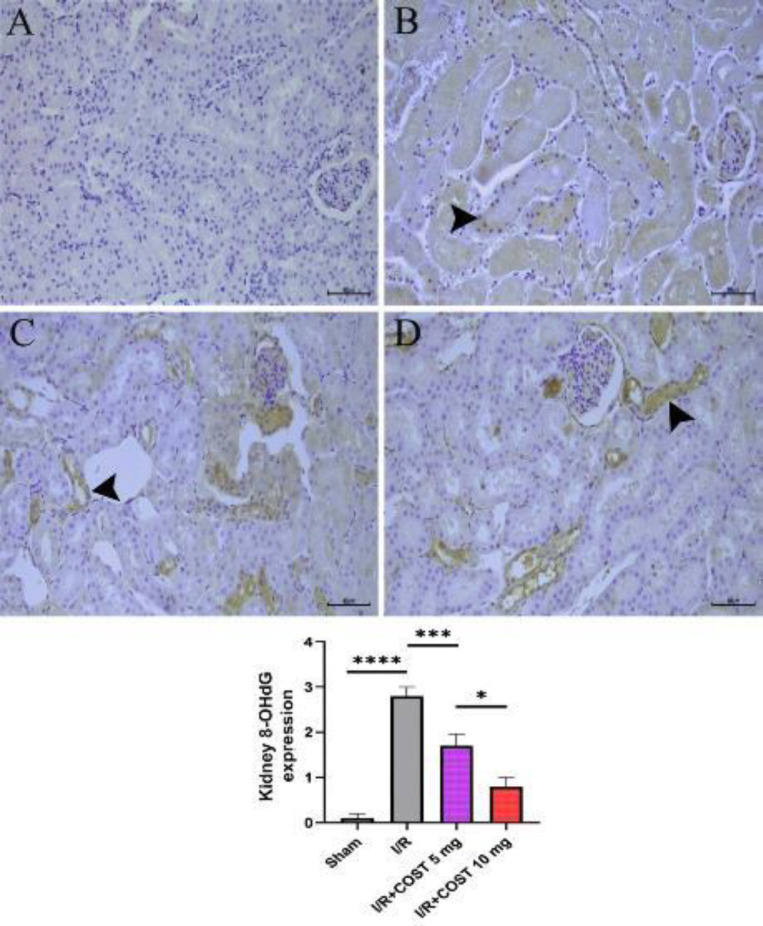
8-OHdG expression and score of 8-OHdG expression in kidney tissues of renal ischemia-reperfusion rat model

Figure 7 depicts the expression and score of 8-OHdG in kidney tissues. In the I/R group (Figure 7B), 8-OHdG expression was higher than in the sham group (*P*<0.05). COST administration reduced the I/R-mediated increase in 8-OHdG expression significantly (*P*<0.05). Furthermore, 8-OHdG expression was considerably lower in the I/R+COST 10 mg/kg group (Figure 7D) than in the I/R+COST 5 mg/kg group (*P*<0.05).

***Caspase-3 (Kidney)***

**Figure 8:**
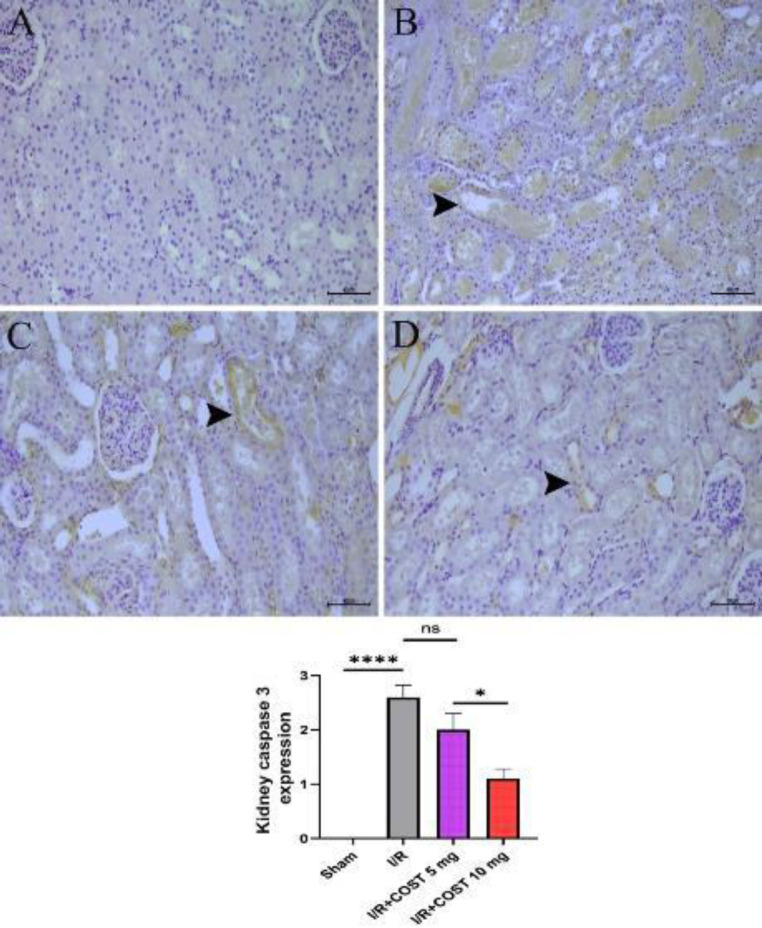
Caspase-3 expression and caspase-3 expression score of kidney tissues renal ischemia-reperfusion rat model

Caspase-3 expression was examined to demonstrate the role of COST in attenuating apoptosis. Figure 8 shows the caspase-3 expression and the caspase-3 expression score determined in kidney tissues. Caspase-3 expression was not detected in the kidney tissue of sham rats (Figure 8A). There was an intense caspase-3 expression in the proximal and distal tubules in the I/R group (Figure 8B). On the other hand, although caspase-3 expression decreased in kidney tissue in the I/R+COST 5 mg/kg group, it did not differ significantly from the I/R group (Figure 8C, *P*>0.05). Caspase-3 expression in the I/R+COST 10 mg/kg group was significantly lower compared to the I/R and I/R+COST 5 mg/kg groups (Figure 8D, *P*<0.05).

***LC3B (Kidney)***

**Figure 9:**
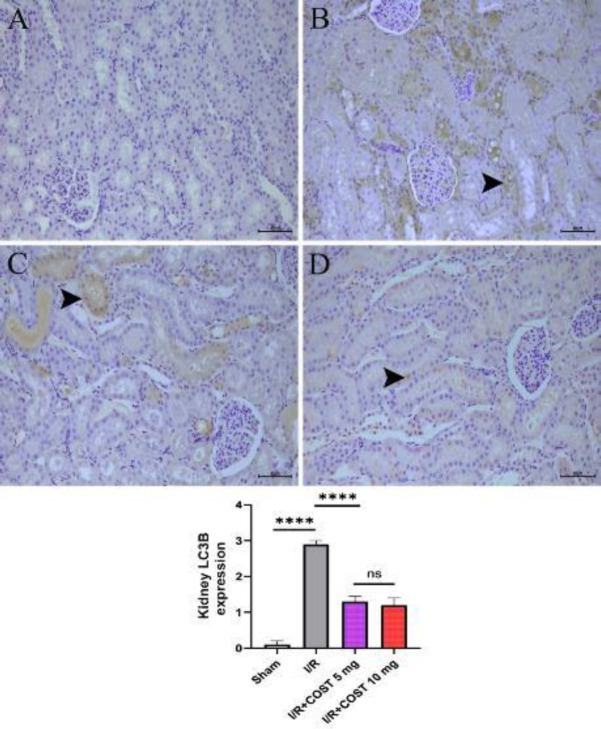
LC3B expression and LC3B expression score of kidney tissues renal ischemia-reperfusion rat model

Figure 9 displays the expression of LC3B and the score of LC3B expression in kidney tissues. Compared to the sham group (Figure 9A), LC3B expression was considerably higher in the I/R group (Figure 9B, *P*<0.05). COST treatment, in contrast, significantly decreased LC3B expression (*P*<0.05, Figures 9C and 9D). Although LC3B expression diminished more in the I/R+COST 10 mg/kg group (Figure 9D) than in the I/R+COST 5 mg/kg group (Figure 9C), there was no noticeable difference between the two groups (*P*>0.05).

***8-OHdG (Lung)***

**Figure 10:**
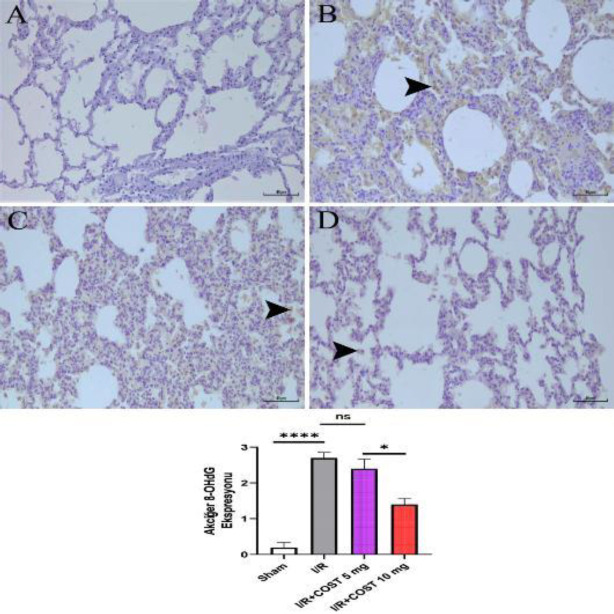
8-OHdG expression and 8-OHdG expression score of lung tissues of renal ischemia-reperfusion rat model

Figure 10 exhibits the expression of 8-OHdG and the score of 8-OHdG expression in lung tissues. The I/R group had higher alveolar cell density with 8-OHdG expression than the sham group (Figures 10B and 10A, *P*<0.05). Although 8-OHdG expression was lower in the I/R+COST 5 mg/kg group than in the I/R group, there was no significant difference (Figures 10C, *P*>0.05). The expression of 8-OHdG was significantly lower in the I/R+COST 10 mg/kg group than in the I/R and I/R+COST 5 mg/kg groups (Figure 10D, *P*<0.05).

***Caspase-3 (Lung)***

**Figure 11:**
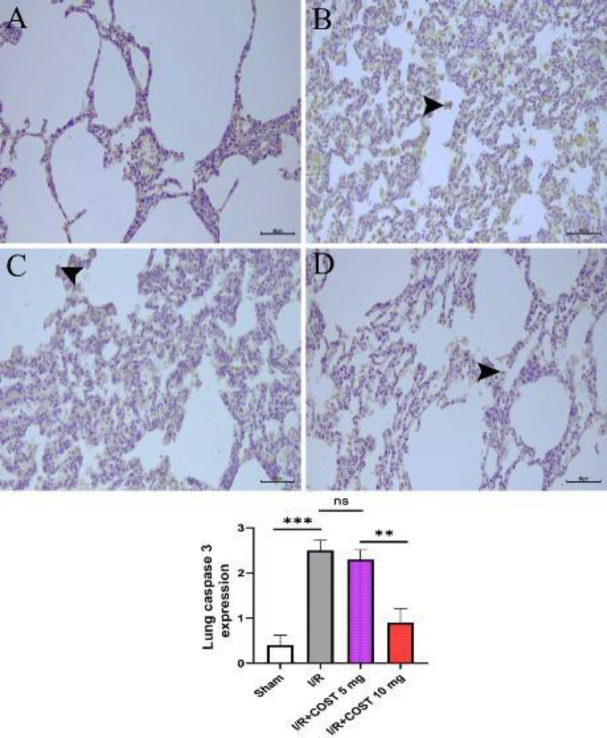
Caspase-3 expression and caspase-3 expression score of lung tissues in the renal I/R model of rats

Figure 11 depicts caspase-3 expression and caspase-3 expression score in lung tissues. Caspase-3 expression was significantly higher in the I/R group’s alveolar cells (Figure 11B) compared to the sham group (Figure 11A, *P*<0.05). Although caspase-3 expression was lower in the I/R+COST 5 mg/kg group, there was no statistically significant difference compared to the I/R group (Figure 11C, *P*>0.05). Caspase-3 expression was significantly lower in the I/R+COST 10 mg/kg group (Figure 11D) than in both I/R and I/R+COST 5 mg/kg groups (*P*<0.05).

***LC3B (Lung)***

**Figure 12:**
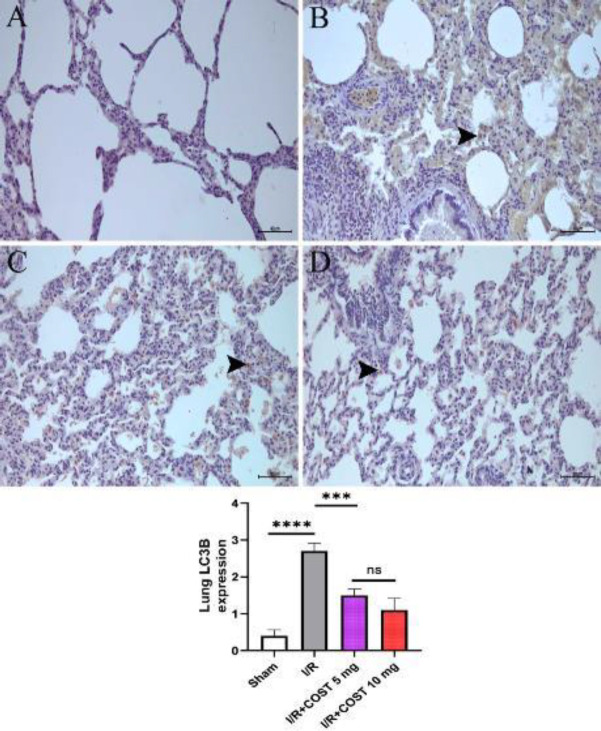
LC3B expression and LC3B expression score of lung tissues renal ischemia-reperfusion rat model

Figure 12 describes the LC3B expression and LC3B expression score in the study’s lung tissues. The I/R group had higher LC3B expression than the sham group (Figures 12B and 12A, *P*<0.05). COST administration significantly reduced LC3B expression in interstitial lung cells (*P*<0.05). Although the I/R+COST 10 mg/kg group (Figure 12D) had lower LC3B expression than the I/R+COST 5 mg/kg group (Figure 12C), there was no significant difference (*P*>0.05).

## Discussion

AKI is a common health problem (41) with a higher risk of death (42). I/R is a major parameter among the AKI factors (43). Several conditions like kidney transplantation and hypovolemic shock cause renal I/R injury (44). I/R-induced injury has no effective treatment, despite many prevention methods (45). Here, we revealed the preventive effects of COST on renal I/R injury via biochemical and immunohistochemical findings. 

In the literature, COST reduced MDA levels significantly in an ethanol-induced gastric ulcer mouse model (46). COST administration also decreased the MDA levels in a lung fibrosis mouse model (47). MPO activity in a colitis-induced mice experiment decreased with COST administration (32). A decrease in MPO activity was noted after COST administration in a mouse model with lung injury (48). Our findings are compatible with this literature. 

In previous research, oxidative stress-related low GSH and SOD levels increased with COST use in a streptozotocin-induced diabetes model in rats (49). Besides, COST increased GSH and SOD levels in a pulmonary fibrosis mouse model (47). Our results are consistent with these data. 

In a mouse study of ethanol-induced gastric ulcers, COST administration declined the TNF-α levels (46). COST reduced the inflammatory response in mice with ulcerative colitis by lowering the proinflammatory cytokines IL-1β, IL-6, and TNF-α (32). In a study of mice with acute liver damage, COST administration reduced IL-1β and TNF-α levels (50). COST also inhibited lipopolysaccharide-induced IL-1β increase in another study  (51). In a mouse model of acute lung injury, COST reduced TNF-α expression (48). COST reduced lung inflammation in an asthma mouse model by inhibiting the expression of TNF-α-induced chemokines (52). In a study of pulmonary fibrosis in mice by Liu *et al*., COST declined IL-6 levels (47). Compared to the examples in the literature, COST demonstrated anti-inflammatory activity and decreased proinflammatory cytokine levels in our study. 

COST inhibited apoptosis by decreasing the caspase-3 activity as a neuroprotective agent (53). Besides, COST significantly diminished caspase-3 expression in a cerebral I/R injury rat model (54). Here, our results indicated that COST was effective against the apoptosis caused by I/R damage.

A traditional medicine complex, including COST, inhibited autophagy via inhibiting LC3 transcription in a hydrogen peroxide-induced apoptosis study (55). We found that COST lowered LC3B levels significantly and was effective against autophagy in I/R damage. 

Hempistepsin A, a sesquiterpene lactone, declined 8-OHdG production and prevented hydrogen peroxide-induced DNA damage (56). Artesune is a sesquiterpene lactone derivate that alleviated lung injury by inhibiting 8-OHdG levels in mice (57). In this study, according to the findings from both kidney and lung tissues, COST diminished the expression of 8-OHdG, which lessened DNA damage. 

(-)-α-Bisabolol, a member of the sesquiterpene family, reduced KIM-1 levels in a renal cytotoxicity study (58) and a renal I/R injury model (59). β-caryophyllene, a sesquiterpene, diminished KIM-1 levels in a rat nephrotoxicity rat model (60). In this study, the application of COST significantly reduced KIM-1 expression.

Statistically, *P<*0.05 was considered significant. Means written with the same letter in the columns are not statistically different in the Duncan test

Statistically, *P<*0.05 was considered significant. Means written with the same letter in the columns are not statistically different in the Duncan test

MDA: Malondialdehyde; MPO: myeloperoxidase; SOD: superoxide dismutase; GSH: glutathione

Statistically, *P<*0.05 value was considered significant. Means written with the same letter in the columns are not statistically different in the Duncan test

MDA: Malondialdehyde; MPO: myeloperoxidase; SOD: superoxide dismutase; GSH: glutathione

A) Sham group, normal glomerulus (thin arrow), distal (thick arrow), and proximal tubules (arrowhead). B) I/R group, severe coagulation necrosis (arrowhead) and bleeding (arrow). C) I/R+COST 5 mg/kg group, tubular epithelium with moderate pycnotic (arrowhead) and karyolitic (thin arrow) nuclei and hydropic degeneration (thick arrow). E) I/R+COST 10 mg/kg group, tubular epithelium with mild pycnotic nuclei (arrowhead), (HE×40µm). Data are shown as mean±SEM. *P, ***P, and ****P<0.05, difference of I/R, I/R+COST 5 mg/kg, and I/R+COST 10 mg/kg groups compared to sham group

A) Sham group, normal alveolar structure. B) I/R group, intense cellular infiltration (arrow), interalveolar edema (star) and hyperemia (arrowhead) in the interalveolar area. C) I/R+COST 5 mg/kg group, moderate interalveolar edema (star) and inflammatory cell infiltration (arrow). E) I/R+COST 10 mg/kg group, mild edema (star) and inflammatory cell infiltration (arrow), (HE×40µm). Data are shown as mean±SEM. **P and ****P<0.05, difference of I/R, I/R+COST 5 mg/kg, and I/R+COST 10 mg/kg groups compared to the sham group

A) Sham group, B) I/R group, C) I/R+COST 5 mg/kg group, D) I/R+COST 10 mg group. Arrowhead indicates KIM-1 positivity in the tubule epithelium, IHCx40 µm. The histogram expresses the density of positive cells in each group, n=10. Data are shown as mean±SEM. Compared to the sham group ****P<0.05 and *P<0.05 compared to the I/R group, *P<0.05 compared to the I/R+COST 5 mg/kg group

A) Sham group, B) I/R group, C) I/R+COST 5 mg/kg group, and D) I/R+COST 10 mg/kg group. Arrowhead represents 8-OHdG positivity in the tubule epithelium, IHCx40 µm. The histogram describes the density of positive cells in each group, n=10. Data are shown as mean±SEM. ****P<0.05 compared to the sham group, ***P<0.05 compared to the I/R group, and *P<0.05 compared to the I/R+COST 5 mg/kg group

A) Sham group, B) I/R group, C) I/R+COST 5 mg/kg group, and D) I/R+COST 10 mg/kg group. Arrowhead indicates caspase-3 positivity in the tubule epithelium, IHCx40µm. The histogram expresses the density of positive cells in each group, n=10. Data are shown as mean±SEM. ****P<0.05 compared to the sham group, ns: no significant differences compared to the I/R group, *P<0.05 compared to the I/R+COST 5 mg/kg group

A) Sham group, B) I/R group, C) I/R+COST 5 mg/kg group, and D) I/R+COST 10 mg/kg group. Arrowhead indicates LC3B positivity in the tubule epithelium, IHCx40µm. The histogram expresses the density of positive cells in each group, n=10. Data are shown as mean±SEM. ****P<0.05 compared to the sham group and I/R group, ns: no significant differences compared to I/R+COST 5 mg/kg group

A) Sham group, B) I/R group, C) I/R+COST 5 mg/kg group, and D) I/R+COST 10 mg/kg group. Arrowhead indicates 8-OHdG inflammatory cell positivity in the interalveolar and alveolar regions, IHCx40 µm. The histogram expresses the density of positive cells in each group, n=10. Data are shown as mean±SEM. ****P<0.05 compared to the sham group, ns: no significant difference compared to the I/R group, *P<0.05 compared to the I/R+COST 5 mg/kg group

A) Sham group, B) I/R group, C) I/R+COST 5 mg/kg group, and D) I/R+COST 10 mg/kg group. Arrowhead indicates caspase-3 positivity in interalveolar and alveolar inflammatory cells, IHCx40µm. The histogram expresses the density of positive cells in each group, n=10. Data are shown as mean±SEM. ***P<0.05 compared to the sham group, ns: no significant difference compared to the I/R group, **P<0.05 compared to the I/R+COST 5 mg/kg group

A) Sham group, B) I/R group, C) I/R+COST 5 mg/kg group, D) I/R+COST 10 mg/kg group. Arrowhead indicates 8-OHdG inflammatory cell positivity in the interalveolar and alveolar regions, IHCx40 µm. The histogram expresses the density of positive cells in each group, n=10. Data are shown as mean±SEM. ****P<0.05 compared to the sham group, ***P<0.05 compared to the I/R group, ns: no significant difference compared to the I/R+COST 5 mg/kg group

## Conclusion

The study demonstrated that COST ameliorated renal I/R-induced injury through anti-oxidant, anti-inflammatory, and antiapoptotic effects. COST was effective against tubular damage and inflammation, autophagy, DNA damage, and apoptosis through KIM-1, LC3B, 8-OHdG, and caspase-3 expression, respectively. In addition, proinflammatory cytokine production and oxidant parameter levels diminished. 

This is the first study in the literature to investigate the effects of COST on renal I/R injury. For this reason, our results may guide future studies. In addition, there may be some suggestions.

COST demonstrated a dose-dependent increase in anti-inflammatory activity. On the other hand, although there was a significant improvement in oxidant and anti-oxidant parameters, there was no dose-related difference.

COST also influenced the immunohistochemical examination but did not create a dose-dependent difference in some values. Therefore, in future studies, different doses may be preferred. Outside of cancer studies, COST should be considered in I/R-related pathologies, particularly renal conditions. In addition, the reliability of the data will increase by using protein analysis methods in advanced studies.

## Authors’ Contributions

MCG, EA, and AT designed the experiments; MCG, AT, SÇ, and YB performed experiments and collected data; MCG, EA, and AT discussed the results and strategy; MCG supervised, directed, and managed the study; MCG, EA, AT, SÇ, and YB approved the final version to be published.

## Conflicts of Interest

The authors reported no potential conflicts of interest. 
